# Identification of novel MYO18A interaction partners required for myoblast adhesion and muscle integrity

**DOI:** 10.1038/srep36768

**Published:** 2016-11-08

**Authors:** Jian-Meng Cao, Xiao-Ning Cheng, Shang-Qi Li, Stefan Heller, Zhi-Gang Xu, De-Li Shi

**Affiliations:** 1 School of Life Sciences, Shandong University, Jinan 250100, China; 2 Department of Otolaryngology-Head & Neck Surgery; Institute for Stem Cell Biology and Regenerative Medicine, Stanford University School of Medicine, Stanford, CA 94305, USA; 3 Sorbonne Universités, UPMC Univ Paris 06, CNRS, Biologie du développement, Institut de Biologie Paris Seine (LBD-IBPS), 75005 Paris, France

## Abstract

The unconventional myosin MYO18A that contains a PDZ domain is required for muscle integrity during zebrafish development. However, the mechanism by which it functions in myofibers is not clear. The presence of a PDZ domain suggests that MYO18A may interact with other partners to perform muscle-specific functions. Here we performed double-hybrid screening and co-immunoprecipitation to identify MYO18A-interacting proteins, and have identified p190RhoGEF and Golgin45 as novel partners for the MYO18A PDZ domain. We have also identified Lurap1, which was previously shown to bind MYO18A. Functional analyses indicate that, similarly as *myo18a*, knockdown of *lurap1, p190RhoGEF* and *Golgin45* by morpholino oligonucleotides disrupts dystrophin localization at the sarcolemma and produces muscle lesions. Simultaneous knockdown of *myo18a* with either of these genes severely disrupts myofiber integrity and dystrophin localization, suggesting that they may function similarly to maintain myofiber integrity. We further show that MYO18A and its interaction partners are required for adhesion of myoblasts to extracellular matrix, and for the formation of the Golgi apparatus and organization of F-actin bundles in myoblast cells. These findings suggest that MYO18A has the potential to form a multiprotein complex that links the Golgi apparatus to F-actin, which regulates muscle integrity and function during early development.

Vertebrate skeletal muscle cells originate from progenitor cells present in the somites, which are segmented structures formed in an anterior to posterior sequence from the posterior presomitic mesoderm^1^. Muscle progenitor cells form elongated and multinucleated myofibers during differentiation. Each myofiber contains large amounts of myofibrils representing the basic functional units of myofibers that are composed of regularly arrayed sarcomeres. In addition, the peripheral muscle cell membrane (sarcolemma) contains actin-binding dystrophin and associated proteins, forming the dystrophin-associated protein complex (DAPC) that plays a key role in connecting myofibrils and ensuring the stable attachment of muscle cells to extracellular matrix (ECM) proteins^2^,^3^,^4^. Mutations in many different components within this complex, including dystrophin and α-dystroglycan (α-DG), disrupt the structural integrity of the sarcolemma and the connections with ECM, impair muscle attachment and cause several forms of muscular dystrophies^4^,^5^,^6^,^7^,^8^, leading to a varied degree of muscular lesions and degeneration.

The sarcomere represents the basic contractile unit of skeletal muscle and is predominantly composed of actin-containing thin filaments and myosin-containing thick filaments^4^. Different myosin proteins constitute a superfamily of molecular motors and can be grouped into conventional and unconventional classes, which all produce force and movement through ATP hydrolysis. Conventional myosins associate into myofibrils through their long coiled-coil tails. Mutations of several skeletal muscle myosin heavy chains (MHCs) are associated with human myopathies. For example, the “autosomal dominant MHC IIa myopathy (E706K)” or “hereditary inclusion body myopathy type 3” is associated with mutations in the conventional muscle *mhc myh2* gene^9^. Unconventional myosins do not form the structure of myofibrils, however, they have been shown to play important roles in the regulation of a wide range of cellular functions, including cell migration, intracellular trafficking, adhesion and cytokinesis^10^, although their implication in muscle cell function remains elusive. At present, there is only a limited number of studies reporting their involvement in myofiber organization and myoblast differentiation^11^,^12^, making this broad field quite open for further exploration.

Myosin18A, also known as MYO18A/MysPDZ, is the only unconventional myosin containing a PDZ domain in its amino-terminal region^13^,^14^, which may play a central role in mediating protein-protein interactions. Indeed, in mammalian cell lines, it has been shown that MYO18A interacts with Lurap1 (Leucine repeat adaptor protein 1) or Lrap35a, and is involved in regulating cell protrusion and migration^15^,^16^, as well as normal Golgi trafficking and morphology^17^. In zebrafish, *myo18a (myo18aa* and *myo18ab*) genes are predominantly expressed at somite borders during early developmental stages. We have previously shown that knockdown of these genes disrupts myofiber integrity and induces myofiber lesions, and compromises the localization of dystrophin, α-DG and laminin at myotome boundaries^11^, suggesting an important role for this unconventional myosin in maintaining muscle cell stability. Nevertheless, the mechanism by which MYO18A functions in this process remains unclear. Interestingly, overexpression of the MYO18A PDZ domain also disrupts myofiber integrity^11^, probably due to competition for binding to MYO18A interaction partners. Thus, it is of interest to identify and analyze the function of MYO18A PDZ domain-interacting proteins for a better understanding of the molecular network maintaining the integrity of embryonic and adult muscles.

The zebrafish embryo has been widely used as an animal model to study the function of human musclular disease genes^18^,^19^,^20^. Mutation or disruption of many components of the myofibrils^21^,^22^,^23^,^24^,^25^,^26^,^27^, and the DAPC components^5^,^6^,^7^,^8^,^28^, leads to muscle defects, which phenocopy various aspects of human muscle disorders. Furthermore, there exists a striking similarity in the structure and organization of the muscle between zebrafish and human. As an example, the zebrafish myosepta connect two adjacent muscle cells and transmit muscular forces through the myotendinous junction, which establishes transmembrane link between cytoskeletal elements anchored to sarcolemma and ECM proteins. Thus, the myosepta are functionally equivalent to the mammalian tendon and serve as attachment sites between muscle fibers of adjacent myotomes^29^,^30^.

Here we report the identification and functional analyses of novel MYO18A interaction partners in muscle integrity during zebrafish early development. By yeast two-hybrid screening and co-immunoprecipitation, we found that the PDZ domain of MYO18A interacts with p190Rho-guanine nucleotide exchange factor (p190RhoGEF) and Golgin45, which represent novel MYO18A interaction partners. Lurap1, a previously characterized protein^15^, was also identified in the screen. We further show that these proteins functionally interact with MYO18A in maintaining myofiber integrity. In cultured myoblasts, inhibition of MYO18A or its interaction partners blocks the formation of the juxtanuclear Golgi apparatus and F-actin organization, and impairs their adhesion on ECM. Thus, our findings uncovered hitherto unrecognized MYO18A-associated proteins necessary for the integrity of embryonic myofibers. These MYO18A partners are required for normal Golgi morphology and trafficking, which play an important role in the correct localization of membrane receptors for ECM, and thus for the stable attachment of myofibers.

## Results

### Identification of novel MYO18A interaction partners

**Figure 1:**
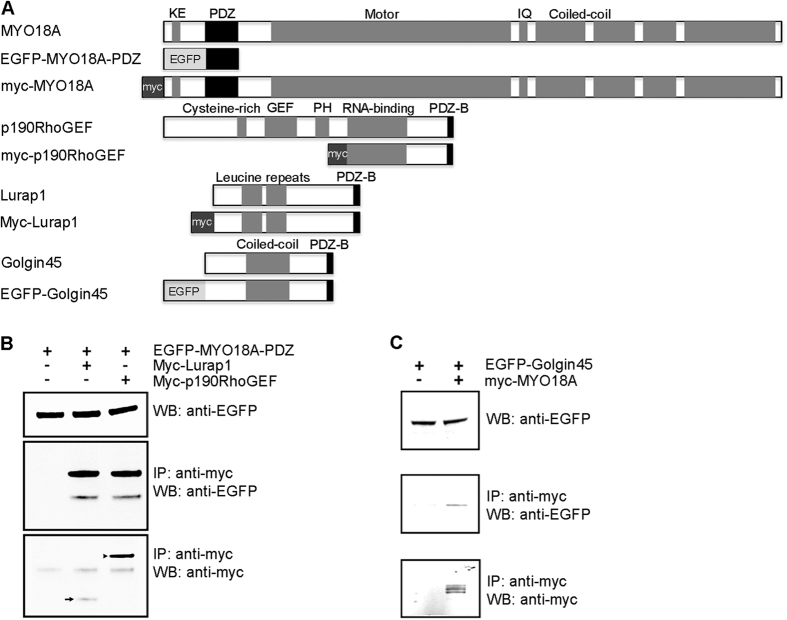
Biochemical interaction between MYO18A and its partners. Co-immunoprecipitation following transfection of different expression vectors in HEK293T cells. (**A**) Schematic representation of full-length proteins and related expression constructs. Known functional domains (solid black or grey boxes) are shown, with abbreviations indicated on the top and in the text. (**B**) EGFP-tagged MYO18A PDZ domain is expressed in different conditions (upper panel) and immunoprecipitated (middle panel) by myc-tagged p190RhoGEF or Lurap1, which are detected by western blot (arrowhead and arrow in lower panel, respectively). (**C**) EGFP-tagged Golgin45 is expressed in different conditions (upper panel) and immunoprecipitated (middle panel) by myc-tagged full-length MYO18A, which is detected by western blot (lower panel).

MYO18A contains a PDZ domain in the amino-terminal region^13^. This feature allows MYO18A to interact with other proteins via its amino-terminus, which is unusual because interactions with other proteins beside F-actin usually happen via the carboxyl-terminal tails of myosins. Furthermore, we have previously found that overexpression of the MYO18A PDZ domain disrupts myofiber integrity^11^, indicating an important role of this region in muscle function. To understand the mechanism by which MYO18A functions in muscle cells, we performed a yeast two-hybrid screening with the goal of identifying novel MYO18A interaction partners. The chicken MYO18A PDZ domain (amino acids 194–338) was used to screen a cDNA library made from chicken inner ear tissue, which is a site with strong MYO18A protein expression (data not shown). A total of 17 clones were isolated, among which 11 clones corresponded to p190RhoGEF, 4 clones matched the basic leucine nuclear factor or JEM-1/BLZF1/Golgin45, and 2 other clones corresponded to Lurap1. Co-immunoprecipitation experiments showed that EGFP-tagged MYO18A PDZ domain was immunoprecipitated by myc-tagged p190RhoGEF carboxyl-terminal region encompassing 951 to 1390 amino acids after the pleckstrin homology (PH) domain (Fig. 1A,B), and EGFP-tagged Golgin45 was immunoprecipitated by myc-tagged full-length MYO18A (Fig. 1A,C). This result confirms the biochemical interaction between MYO18A and p190RhoGEF and Golgin45, and indicates that p190RhoGEF and Golgin45 are novel protein-binding partners of MYO18A. It was shown previously that, in HeLa cells, MYO18A interacts with Lurap1, which contains a class I PDZ-binding (PDZ-B) motif (T-F-L) at its carboxyl-terminus^15^, we also confirmed the interaction by co-immunoprecipitation (Fig. 1B). Sequence analyses revealed that the carboxyl-terminus of p190RhoGEF and Golgin45 possesses a class II PDZ-binding motif (V-Y-L and I-A-L, respectively), which not only supports the presumed interaction between the MYO18A PDZ domain and these proteins, but also suggests that the PDZ domain of MYO18A is able to interact with two distinct classes of PDZ-binding motifs. Our yeast two-hybrid screening thus identified novel interaction partners of MYO18A.

### Somitic expression of MYO18A and its interaction partners

**Figure 2:**
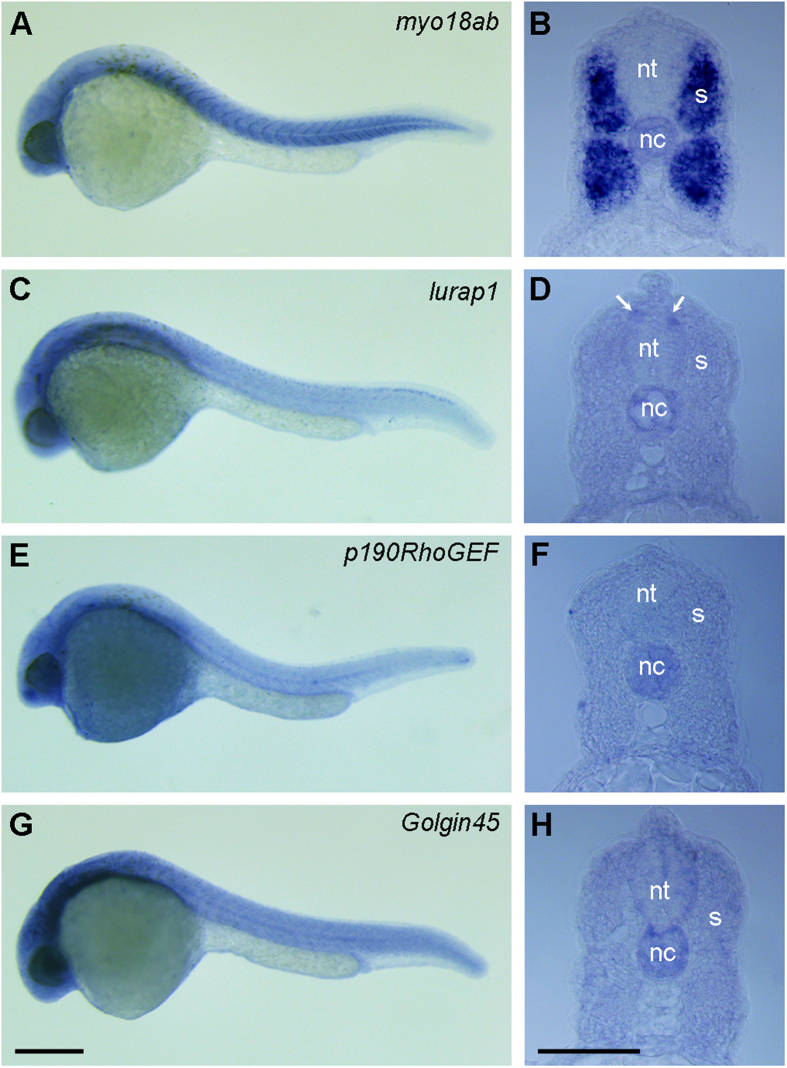
Analysis by *in situ* hybridization of *myo18ab, lurap1, p190RhoGEF* and *Golgin45* expression pattern at 24 hpf. (**A,B**) Expression of *myo18ab* is localized at somite borders and in the somites. Diffuse expression can be observed in the head region. It is strongly localized in deep muscle and weakly in superficial muscle and in the notochord. (**C,D**) Expression of *lurap1* is mainly localized to the head region and weakly in trunk and posterior somites. A punctate pattern can be detected in the dorsal region of the neural tube (arrows). It is also detected in the notochord. (**E,F**) Expression of *p190RhoGEF* can be detected in the head region, in trunk and posterior somites, and in the notochord. Hybridization signal can be also observed in the yolk sac. (**G,H**) Expression of *Golgin45* is localized to the head region, and is also detected in the entire somites and notochord. nt, neural tube; nc, notochord; s, somites. Scale bars: (**A,C,E,G**) 350 μm; (**B,D,F,H**) 50 μm.

We first determined the expression pattern of the four genes by *in situ* hybridization both in whole embryos at 24 hpf (hours post-fertilization), and in axial sections to distinguish the localization of these transcripts in slow and fast muscles^31^. Examination of more than 50 embryos hybridized with each probe from two independent experiments indicated that, in all these embryos, the expression of these genes could be detected in the somites. As previously reported and as *myo18aa*^11^, expression of *myo18ab* was detected in the somites with strong hybridization signal at somite borders. Diffuse expression was also evident in the head region (Fig. 2A). Analysis in histological sections showed that *myo18ab* was most strongly expressed in deeply located fast muscle cells and weakly in the superficial slow muscle cells (Fig. 2B). No expression could be detected in the neural tube, but obvious hybridization signal was present in the notochord (Fig. 2B). Although the expression of *lurap1, p190RhoGEF* and *Golgin45* was relatively ubiquitous, it was still evident in the entire somites, as observed in whole embryos (Fig. 2C,E,G) and in histological sections (Fig. 2D,F,H). The expression of *lurap1* could be also observed in discrete sites in the dorsal region of the neural tube (Fig. 2C,D). In addition, as *myo18ab*, all three other genes were also evidently expressed in the notochord (Fig. 2D,F,H). Thus, the expression in the somites and in the notochord suggests an overlapping expression pattern between MYO18A and its interacting proteins.

### Functional requirement of MYO18A and its interaction partners in myofiber integrity

**Figure 3:**
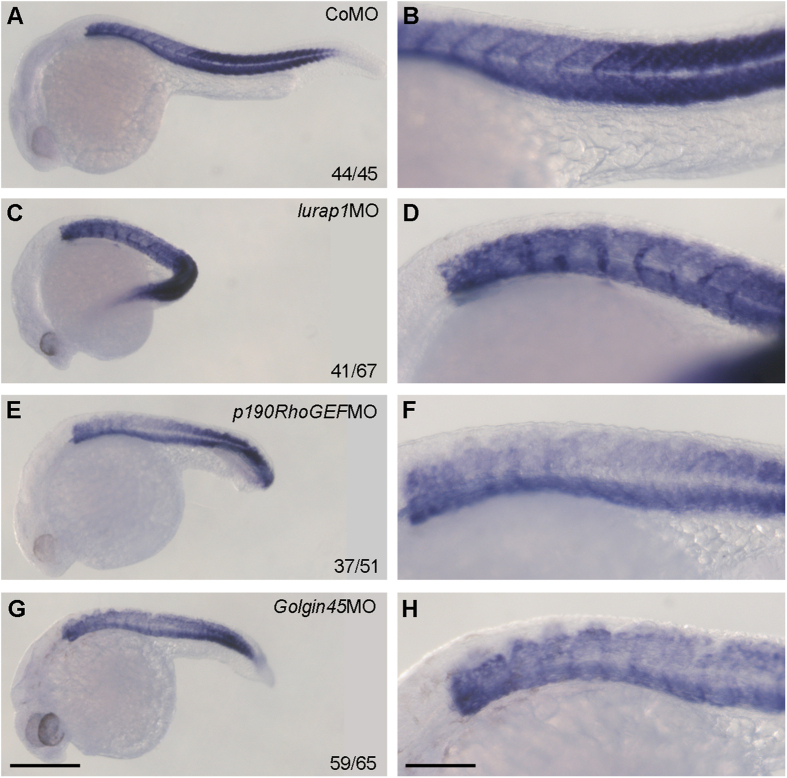
Expression pattern of muscle-specific *mhc* in representative control and morphant embryos at 24 hpf. (**A,B**) A CoMO-injected embryo showing regular *mhc* expression pattern in the somites. (**C,D**) A *lurap1*MO-injected embryo with bent axis and disrupted *mhc* expression pattern in the somites. (**E,F**) A *p190RhoGEF*MO-injected embryo with shortened anteroposterior axis and absence of *mhc* expression at somite boundaries. (**G,H**) A *Golgin45*MO-injected embryo with reduced anteroposterior axis associated with disrupted *mhc* expression pattern in the somites. Scale bars: (**A,C,E,G**) 350 μm; (**B,D,F,H**) 120 μm.

Lurap1 as well as RhoGEF play an important functional role in dynamic organization processes of the actin cytoskeleton^15^,^32^,^33^. Golgin45 is necessary for tethering events in membrane fusion and as structural supports for the Golgi cisternae^34^. We hypothesized that interaction of these proteins with MYO18A indicates that Lurap1, p190RhoGEF and Golgin45 are required for myofiber integrity. To analyze their function, we performed knockdown of these genes using *myo18aa* and *myo18ab* splicing-blocking MOs (*myo18aa*MO and *myo18ab*MO), *lurap1* translation-blocking MO (*lurap1*MO), *p190RhoGEF* splicing-blocking MO (*p190RhoGEF*MO), or *Golgin45* translation-blocking MO (*Golgin45*MO), and examined myofiber organization. When the phenotypes were examined at 48 hpf, embryos injected with 20 ng control MO (CoMO) were normal with clear appearance of somite borders (Supplementary Fig. 1). In contrast, like *myo18aa* or *myo18ab* mrophants^11^, those embryos injected with 20 ng *lurap1*MO, *p190RhoGEF*MO or *Golgin45*MO exhibited shortened and bent anteroposterior axis, small heads and eyes with a varying degree of severity and in a dose-dependent manner (Supplementary Fig. 1). Mildly affected embryos showed a weak shortening of anteroposterior axis, and moderately affected embryos exhibited ventrally bent trunk and tail regions, while severely affected embryos were even more curved with strongly reduced or absence of yolk extension (Supplementary Fig. 1). At 96 hpf, examination by differential interference contrast microscopy revealed that, in CoMO-injected embryos, the myosepta were clearly visible and myofibers were regularly aligned with clear sarcomere structures (Supplementary Fig. 2). In contrast, *lurap1*MO-, *p190RhoGEF*MO- or *Golgin45*MO-injected embryos exhibited irregular or disrupted myosepta and disorganized myofibers with absence of sarcomere structures (Supplementary Fig. 2), as we have previously observed in *myo18aa*MO- and *myo18ab*MO-injected embryos^11^. Analysis by whole-mount *in situ* hybridization of the expression pattern of muscle-specific *mhc* gene further indicated that somites were disorganized in these morphant embryos (Fig. 3). These observations suggest that knockdown of *lurap1, p190RhoGEF* or *Golgin45* disrupts myofiber organization in the zebrafish embryo.

**Figure 4:**
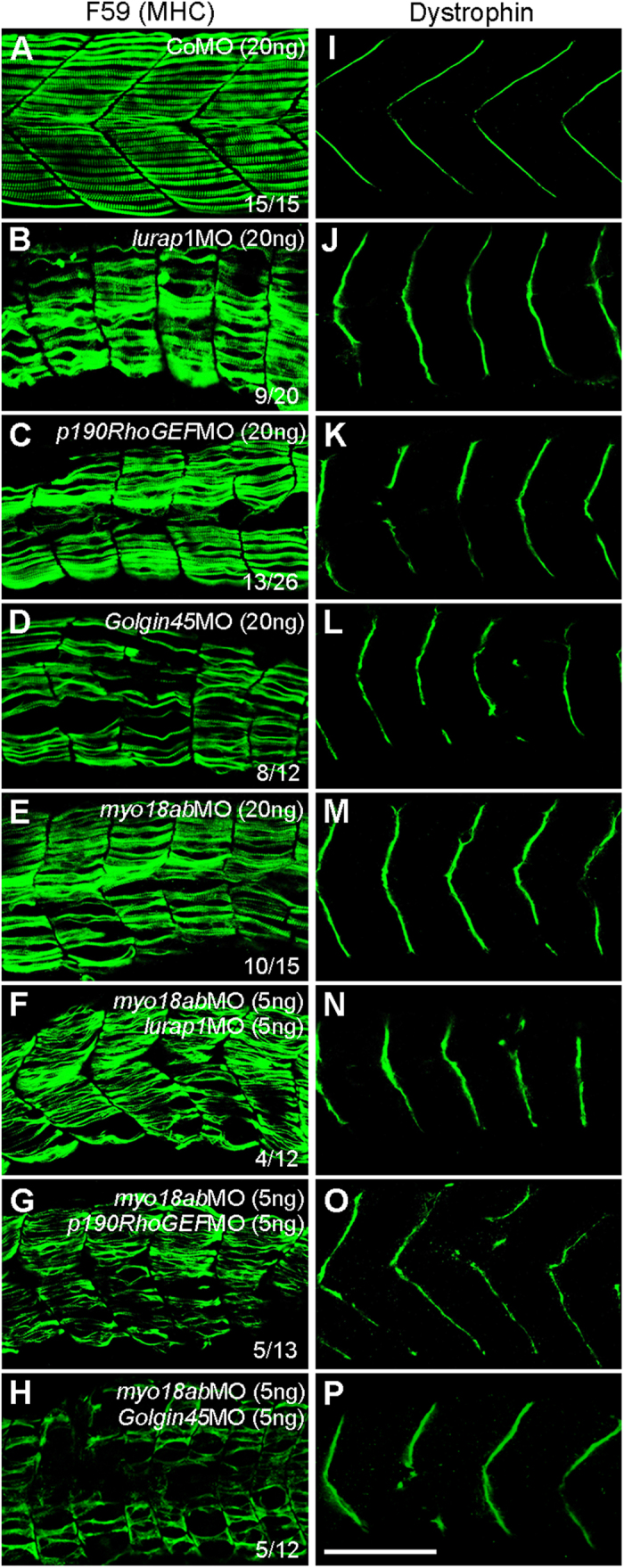
Functional interaction between MYO18A, Lurap1, p190RhoGEF and Golgin45 in myofiber integrity and dystrophin localization. Representative images showing the immunostaining results of slow muscle-specific MHC (**A–H**) and dystrophin (**I–P**) in control and various morphant embryos, as indicated. (**A**,**I**) Regular organization of myofibers within the somite (**A**) and localization of dystrophin at the sarcolemma (**I**) in CoMO-injected embryos, with clear chevron-shape myosepta. (**B–E, J–M**) Individual knockdown using high amounts of MOs against *lurap1* (**B,J**), *p190RhoGEF* (**C,K**), *Golgin45* (**D,L**) or *myo18ab* (**E,M**) produces similar muscle lesions (**B–E**) and disrupts dystrophin localization (**J–M**) at the sarcolemma. (**F–H, N–P**) Simultaneous knockdown using low amounts of indicated MOs also affects myofiber integrity (**F–H**) and dystrophin localization (**N–P**), with severely disorganized myofibers and strongly disrupted myosepta in a high proportion of morphant embryos. Scale bar: 100 μm.

To better examine these myofiber defects, we used F59 antibody to stain MHC of the slow muscles, and dystrophin antibody to stain somite boundaries. Analyses by confocal microscopy indicated that, in CoMO-injected embryos at 28 hpf, myofibers were regularly aligned within each somite (Fig. 4A), and dystrophin protein was localized to myotome boundaries resulting in a regular immunostaining pattern of fine chevrons (Fig. 4I). However, myofiber disorganization (Fig. 4B–D) and disrupted dystrophin localization (Fig. 4J–L) were evident in *lurap1, p190RhoGEF* or *Golgin45* morphants, in a similar manner as in embryos injected with *myo18ab*MO (Fig. 4E,M). Notably, there are gaps within the somites associated with disorganized myofibers. The dystrophin expression pattern was also irregular and occasionally disrupted at somite borders. To examine whether there is a functional interaction between MYO18A and its partners, we coinjected *myo18ab*MO along with *lurap1*MO, *p190RhoGEF*MO or *Golgin45*MO. The results showed that single injection of a low amount of each of these MOs (5 ng) produced essentially normal or mildly affected phenotypes (Supplementary Fig. 1) and did not disrupt muscle integrity in a majority of embryos (Supplementary Fig. 3). However, simultaneous knockdown of *myo18ab* and *lurap1a, myo18ab* and *p190RhoGEF* or *myo18ab* and *Golgin45* resulted in more severely affected phenotypes (Supplementary Fig. 1) and disorganized myofibers (Fig. 4F–H). As expected, dystrophin localization at somite borders was also severely disrupted (Fig. 4N–P). These observations, when combined with the results from protein interaction experiments, suggest that MYO18A may function together with Lurap1, p190RhoGEF and Golgin45 in maintaining the correct localization of dystrophin and myofiber organization.

**Figure 5:**
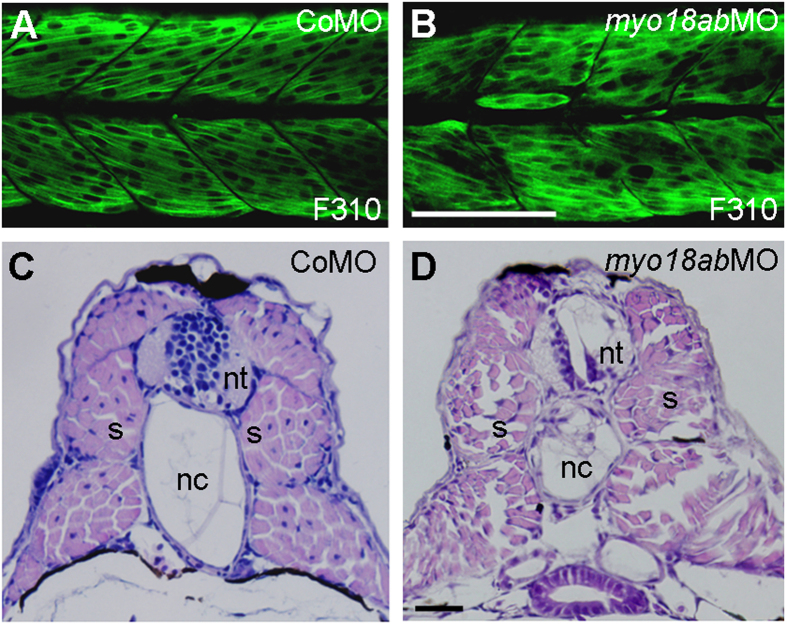
Knockdown of *myo18ab* disrupts the integrity of deeply located fast muscle cells. (**A,B**) Immunofluorescence staining of fast myofibers by F310 antibody in CoMO- and *myo18ab*MO-injected embryos. (**C,D**) Transverse histological sections showing the organization of deeply located fast muscle cells in control and *myo18ab*MO-injected embryos at 96 hpf. Notice that the control embryo has myofibers with centrally located nuclei, while the morphant embryo shows disorganized myofibers with nuclei located at the periphery. nt, neural tube; nc, notochord; s, somite. Scale bars: (**A,B**) 100 μm; (**C,D**) 50 μm.

Skeletal muscle in zebrafish is composed of superficially located slow-twitch and deeply located fast-twitch muscles^35^. Fast muscles are syncytial fibers that constitute the bulk of the myotome and can be labelled by F310 antibody against fast muscle-specific myosin light chain (MLC). Immunofluorescence staining using F310 antibody indicated that, in CoMO-injected embryos at 28 hpf, multinucleated fast myofibers were regularly aligned within the somites, with individual myofiber readily visible (Fig. 5A), while they were clearly irregular in *myo18ab*MO-injected embryos at the same stage (Fig. 5B). Analysis in transverse histological sections further confirmed this result. It showed that control embryos at 96 hpf displayed well-organized deep muscle cells, which had centrally located nuclei (Fig. 5C). In contrast, deep muscle cells in *myo18ab*MO-injected embryos at the same stage were disorganized and the nuclei were located in the periphery of myofibers (Fig. 5D). The same result was also observed in *lurap1*MO-, *p190RhoGEF*MO- or *Golgin45*MO-injected embryos (Supplementary Fig. 4). By randomly choosing one section from an embryo and counting all the cells with a nucleus, we found that 85% of cells counted from all control embryos (*n* = 21) had centrally located nuclei, while 95% of cells from all *myo18ab* morphant embryos examined (*n* = 14) showed peripherally located nuclei. This indicates that MYO18A and its interaction partners are also required for the integrity of fast myofibers.

Using a touch-stimulated “escape response” test, we found that *lurap1* or *p190RhoGEF* morphants at 72 hpf either moved a shorter distance or just turned around upon stimulation (Supplementary Fig. 5), indicating that knockdown of these genes produced embryos with locomotion defects. Since knockdown of *myo18a*, either *myo18aa* or *myo18ab*, also affected the movement ability^11^, these results indicate that MYO18A and its associated proteins function similarly in embryonic muscle cells.

### MYO18A and its interaction partners are required for adhesion of myoblasts to ECM

Lurap1 and p190RhoGEF regulate adhesion-mediated cell motility^15^,^36^, and Golgin45 participates in the secretory protein transport^37^. All these processes are related to cytoskeletal organization. MYO18A binds to actin, and the physical and functional interaction of Lurap1, p190RhoGEF and Golgin45 with MYO18A raises the possibility that the disrupted localization of dystrophin at somite borders may be a consequence of disrupted cytoskeletal actin, which is bound by dystrophin and transmits the mechanical force through DAPC to ECM. As a result, inhibiting the function of MYO18A and its interaction partners should affect the adhesive activity of muscle cells. We thus examined the ability of different morphant myoblast cells to adhere on an ECM component.

**Figure 6:**
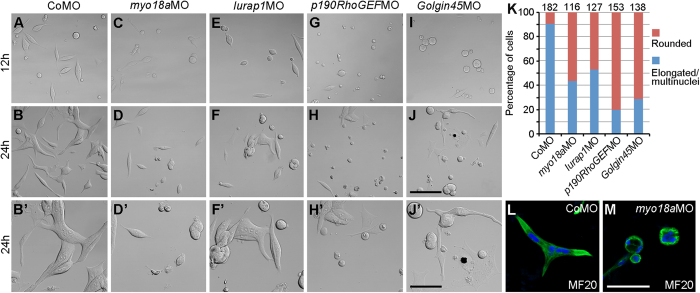
MYO18A and its interaction partners are required for adhesion of muscle cells on ECM. Myoblasts were taken from the somites of indicated control or morphant embryos at 24 hpf, and cultured on laminin substrate for 12 and 24 hours. (**A-B’**) CoMO-injected myoblasts efficiently adhere and extensively elongate to form multinucleated cells on laminin-coated substrate. (**C-D’**) *myo18a (myo18aa* and *myo18ab*) morphant myoblasts exhibit strongly reduced adhesion at 12 hours, and decreased ability to form elongated and multinucleated myofibers at 24 hours. (**E-F’**) *lurap1* morphant myoblasts adhere less efficiently to laminin-coated substrate at 12 hours, and elongate at a reduced extent. (**G-H’**) *p190RhoGEF*MO severely impairs myoblast adhesion, morphology and formation of elongated myofibers. (**I-J’**) *Golgin45*MO strongly affects myoblast adhesion at 12 hours, with few cells elongated at 24 hours. (**K**) Statistical analysis of elongated myoblast cells. Numbers on the top of each column indicate total cells scored from two independent experiments. (**L,M**) Positive MF20 immunostaining of cultured myoblast cells from CoMO- and *myo18a*MO-injected embryos. Scale bars: (**A–J**) 100 μm; (**B’,D’,F’,H’,J’**) 50 μm; (**L,M**) 50 μm.

Somites from control and different morphant embryos at 24 hpf were dissected and dissociated into single cells. They were then cultured on a laminin-coated substrate for different periods. Myoblast cells derived from CoMO-injected embryos rapidly adhered to the substrate. At 12 hours of culture, most cells already exhibited a fibroblastic shape (Fig. 6A). When cultured for 24 hours, they formed multinucleated and strongly elongated myoblasts, showing the characteristic features of myofibers (Fig. 6B,B’). In contrast, a majority of cells derived from embryos previously injected with an equal amount (10 ng) of *myo18aa*MO and *myo18ab*MO (*myo18a*MO), and 20 ng *lurap1*MO, *p190RhoGEF*MO or *Golgin45*MO did not efficiently adhere to the substrate, and took a rounded shape after 12 hours of culture (Fig. 6C,E,G,I). When they were cultured for 24 hours, most morphant cells kept a rounded shape although they were adherent to the substrate (Fig. 6D,D’’). Only few cells were found elongated with some features of multinucleated myofibers (Fig. 6K). By immunofluorescence staining, we checked that both elongated control cells and rounded *myo18a* morphant cells were positively stained by MF20 antibody against muscle myosin II (Fig. 6L,M), suggesting that they were indeed myoblasts. Since laminin is a ligand of α-DG in the DAPC complex, this result indicates that knockdown of these genes reduces the ability of myoblasts to adhere to ECM and impairs the differentiation of myofibers.

### Knockdown of *myo18a, lurap1, p190RhoGEF* and *Golgin45* disrupts the formation of the Golgi apparatus

A previous study has suggested that MYO18A co-localizes with ER-Golgi^14^. In addition, MYO18A, by connecting the Golgi apparatus to F-actin through the direct interaction with GOLPH3, it participates in its vesiculation and contributes to the Golgi’s flattened morphology, which is necessary for normal trafficking^17^. This raises the possibility that the disrupted localization of dystrophin at somite borders might be a consequence of disrupted Golgi apparatus, which regulates trafficking of α-DG to the plasma membrane^38^. We thus examined the formation of the Golgi apparatus in cultured myoblast cells, which offered a better resolution for the detection of the Golgi apparatus, as well as F-actin organization.

**Figure 7:**
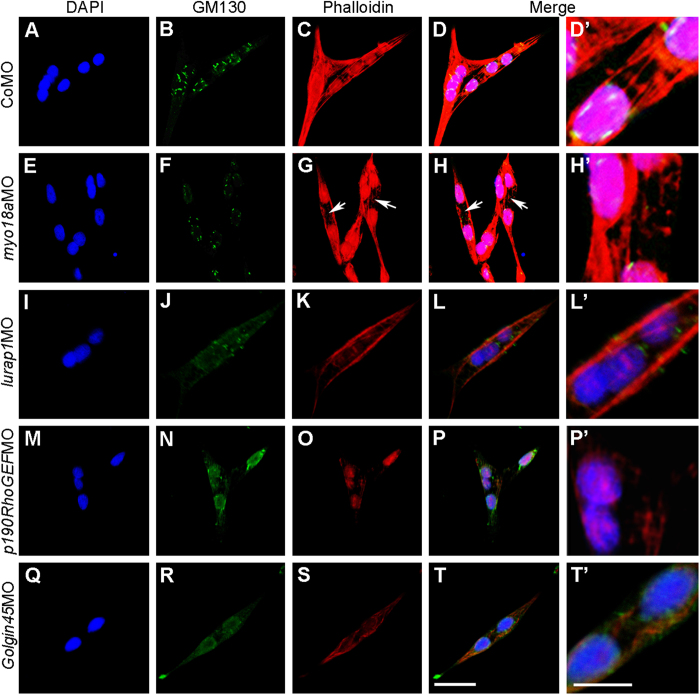
MYO18A and its interaction partners are required for maintaining the morphology of the Golgi and the organization of F-actin network. Staining of the Golgi apparatus by GM130 antibody, F-actin by TRITC-phalloidin and cell nuclei by DAPI was performed in myoblasts cultured for 24 hours in laminin-coated substrate. (**A-D’**) Localization of the juxtanuclear cis-Golgi and the organization of F-actin bundles in CoMO-injected cells. (**E-H’**) Knockdown of *myo18a (myo18aa* and *myo18ab*) reduces the intensity of GM130 labelling in juxtanuclear cis-Golgi and leads to a disrupted F-actin organization (arrows in **G**,**H**). (**I-L’**) Knockdown of *lurap1* leads to diffuse GM130 staining and disrupts F-actin bundles within the cells, resulting in phalloidin staining accumulated at the periphery. (**M-P’**) Diffuse GM130 staining and strongly reduced F-actin bundles in *p190RhoGEF* morphant myoblast cells. (**Q-T’**) Knockdown of *Golgin45* severely reduces the formation of the Golgi apparatus and F-actin bundles. Scale bars: (**A–D,E–H,I–L,M–P,Q–T**) 20 μm; (**D’,H’,L’,P’T’**) 10 μm.

Myoblast cells were dissociated and cultured as above. After 24 hours of culture, confocal microscopic analysis following GM130 staining of the cis-Golgi matrix was performed to monitor the presence of the Golgi apparatus in control and different morphant cells. DAPI staining served to label the nuclei and TRITC-phalloidin staining was used to examine F-actin organization. More than 30 multinucleated cells from two independent experiments were examined in each condition. The results showed that intense juxtanuclear GM130 staining of the vesiculated Golgi was observed in CoMO-injected cells, which present the features of multinucleated and elongated myofibers (Fig. 7A,B). These cells also showed regularly aligned F-actin bundles (Fig. 7C-D’). In all *myo18a*MO-injected cells, however, there was a marked decrease in the intensity of GM130 staining associated with an apparent reduction of the size of the vesiculated Golgi complex (Fig. 7E,F). In addition, although these cells were apparently multinucleated and elongated, there was a clear disruption of the F-actin network (Fig. 7G-‘H). We thus conclude that MYO18A is required for normal Golgi formation and trafficking in myoblasts.

Since Lurap1 and p190RhoGEF physically and functionally interact with MYO18A, we further examined whether knockdown of these genes also affects Golgi morphology. In all cells examined, it appeared that knockdown of *lurap1* resulted in reduced vesiculated Golgi apparatus, as revealed by GM130 staining that was diffusely distributed in the cells (Fig. 7I,J). F-actin bundles were strongly reduced or absent and phalloidin staining was mainly located at the cell periphery (Fig. 7K-L’). Similarly, knockdown of *p190RhoGEF* led to a diffuse juxtanuclear GM130 staining, with the vesiculated Golgi apparatus strongly reduced or nearly absent (Fig. 7M,N). Most obviously, there was a weak phalloidin staining and an absence of F-actin bundles in these cells (Fig. 6O-P’). In all *Golgin45* morphant cells examined, GM130 staining was dramatically reduced and became diffusely distributed around the nucleus, even in elongated and multinucleated cells (Fig. 7Q,R). There was no apparent formation of F-actin bundles and phalloidin staining was also accumulated at the cell periphery (Fig. 7S-T’). These results suggest that MYO18A and its interaction partners are required for normal Golgi morphology in myoblasts and that they may function in regulating trafficking of dystrophin essential for myoblast attachments and integrity.

## Discussion

In the present study, we have identified novel interaction partners of the MYO18A PDZ domain. Our findings suggest that both MYO18A and its interaction partners including Lurap1, p190RhoGEF and Golgin45 function to regulate the normal morphology and trafficking of the Golgi apparatus. They are required for F-actin organization in the myofibers and for the correct localization of dystrophin at somite borders, which in turn may affect the attachment of myofibers to ECM.

MYO18A is a unique unconventional myosin that possesses a PDZ domain^13^,^14^, suggesting that it has the potential to function as a multiprotein complex in different biological processes. Indeed, PDZ domains are protein interaction modules that often recognize short amino acid motifs that are most commonly located at the carboxyl-termini of target proteins. They regulate multiple biological processes such as transport, ion channel signalling, and other signal transduction pathways through interaction with other proteins^39^. It has been shown that MYO18A interacts with the adaptor protein Lurap1 to regulate cell migration in cultured cells^15^. We have previously shown that, in zebrafish, knockdown of *myo18aa* and *myo18ab* disrupted the localization of dystrophin and α-DG at somite borders, and produced myofiber lesions^11^. Furthermore, overepexression of the MYO18A PDZ domain also produced muscle cell lesions, similarly as in morphant embryos^11^. This suggests that the PDZ domain of MYO18A plays an important role in maintaining the integrity of myofibers. However, at present, Lurap1 is the only protein that has been shown to interact with the MYO18A PDZ domain in cultured cells^15^, although GOLPH3 has been also shown to interact with the N-terminal and middle fragments of MYO18A^17^. In this work we have identified two novel protein partners that interact with the PDZ domain of MYO18A. Both yeast two-hybrid screening and co-immunoprecipitation indicate that the PDZ domain of MYO18A physically interacts p190RhoGEF and Golgin45, which contain a class II PDZ-binding motif at their carboxyl-termini. Lurap1 contains a class I PDZ-binding motif at its carboxyl-terminus, which is required for interaction with MYO18A^15^. This observation combined with our results suggest that the PDZ domain of MYO18A is able to bind to distinct classes of PDZ-binding motifs and may be involved in the regulation of multiple biological processes.

**Figure 8:**
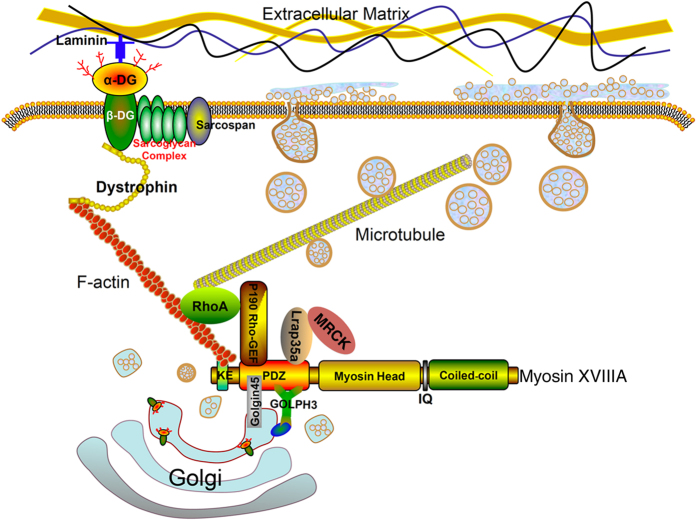
A model of MYO18A function in maintaining the stability of myofibers. MYO18A is located at the Golgi apparatus through interaction of its PDZ domain with GOLPH3 and Golgin45. It also directly binds F-actin, which is connected to dystrophin. The interaction between MYO18A and p190RhoGEF, as well as Lurap1, regulates the organization of cytoskeleton and Golgi-mediated trafficking. Thus, MYO18A may link the Golgi apparatus to dystrophin through F-actin.

We also showed that MYO18A functionally interacts with these proteins and our experimental evidence suggests that they are all involved in regulating myofiber integrity since individual knockdown of these genes produces similar myofiber defects and simultaneous knockdown has a stronger effect. A previous study showed that MYO18A interacts with Lurap1 to regulate cell protrusion and migration^15^. It is also well established that RhoGEF is involved in the dynamic organization of the actin cytoskeleton through regulation of the Rho family of small GTPases^32^,^33^. Accordingly, knockout of *p190RhoGEF* in mice inhibits RhoA activity and adhesion-mediated cell motility^36^. These studies provided some clues about the mechanism by which MYO18A could function in myofiber integrity. Based on our results and consistent with the binding of MYO18A to actin filaments^40^,^41^, it is possible that MYO18A could also function as multiprotein complex involved in regulating the organization of actin cytoskeleton at interaction points with the plasma membrane, as depicted in the model (Fig. 8). Because F-actin plays an essential role for the attachment of myofibers to ECM through interaction with dystrophin and α- and β-DG^2^, it is likely that inhibition of the function of MYO18A and its associated proteins results in myofiber lesions through disruption of actin cytoskeleton.

In addition to affecting actin cytoskeleton, MYO18A and its interaction partners may also regulate other cellular aspects that could affect myofiber attachment to ECM, rendering the mechanism(s) of action more complicated. A previous study has functionally linked the Golgi protein GOLPH3 with MYO18A, and showed that this interaction is required for the Golgi localization of MYO18A and normal Golgi morphology^17^. Our yeast two-hybrid screen identified Golgin45, a coiled-coil protein associated with the Golgi apparatus necessary for tethering events in membrane fusion and as structural supports for the Golgi cisternae^34^. These findings are consistent with the requirement of the PDZ domain for MYO18A localization in the ER-Golgi^14^ and they suggest that both GOLPH3 and Golgin45 could be involved in this subcellular localization. In support of this conclusion, we have previously shown that overexpression of the MYO18A PDZ domain also disrupts myofiber integrity^11^. Since all the MYO18A interaction partners that we identified interact with its PDZ domain, it is not surprising that the overexpressd MYO18A PDZ domain should sequester these proteins and prevent MYO18A-mediated connection of Golgi to F-actin. Nevertheless, we cannot exclude the possibility that the overexpressed MYO18A PDZ domain could interfere with other pathways. For example, the non-canonical Wnt pathway, which utilizes the PDZ domain-containing Dishevelled protein, was shown to be required for myofiber elongation in amniotes^42^. Given the importance of Dishevelled and the non-canonical Wnt pathway in regulating cytoskeleton, cell adhesion and morphogenetic movements^43^, it raises the intriguing question whether MYO18A and the non-canonical Wnt pathway interact or converge in myoblast cell adhesion.

We find that knockdown of *myo18a* disrupts the formation and morphology of the Golgi apparatus in cultured myoblast cells. Knockdown of *lurap1, p190RhoGEF* and *Golgin45* has both similar and distinct effects on the formation of the juxtanuclear cis-Golgi. In all these morphant cells, there is a clear disorganization of F-actin network. Our present observation, complements with a previous study showing that GOLPH3 bridges MYO18A to the Golgi apparatus^17^, and further highlights the importance of MYO18A in connecting the Golgi to F-actin (Fig. 8). Because of this key position and function, inhibition of MYO18A or its interaction partners disrupts normal Golgi morphology and could impair trafficking of membrane-associated or extracellular proteins from the Golgi to the plasma membrane. This could explain, at least partially, the disrupted localization of the DAPC component, dystrophin, at the sarcolemma of different morphant embryos. Consistent with this conclusion, it has been shown that depletion of Golgin45 disrupts the Golgi apparatus and causes a block in secretory protein transport^37^. Furthermore, a causal relationship exists between microtubule stability and Golgi organization and trafficking. For example, loss-of-function of dystonin cytoskeletal linker proteins impairs these processes^44^. In the present study, we find that inhibition of the function of MYO18A and its interaction partners disrupts the organization of F-actin bundles and normal Golgi morphology in myoblast cells. Thus, we could speculate that MYO18A may function as a multivalent scaffold for both Golgi-associated proteins and factors regulating cytoskeleton.

Golgi-mediated posttranslational modifications also play important roles in the functional specialization and trafficking of proteins^38^,^45^. For example, the identification of mutations linked to defective glycosylation of α-DG has shed light on a novel mechanism responsible for muscular dystrophies^46^. These mutations affecting the enzymatic activity or the localization in the Golgi or endoplasmic reticulum of several proteins cause muscle degeneration associated with hypoglycosylation of α-DG in humans and in zebrafish^47^,^48^,^49^,^50^,^51^, suggesting a conserved requirement of Golgi function in maintaining muscle integrity. Our results suggest that MYO18A and its interaction partners have the potential to form a multiprotein complex required for Golgi function in muscle cells. However, MYO18A may also interact sequentially with these partners and regulates distinct aspects related to myoblast adhesion to ECM. Since functional inhibition of either of these proteins leads to myofiber disorganization and reduced adhesion on laminin, a ligand for α-DG, there is a possibility that the interaction of MYO18A with its partners may be indirectly involved in α-DG glycosylation through regulation of Golgi formation and trafficking. Thus, it will be of interest to examine whether MYO18A affects the glycosylation status of α-DG in myoblasts, thereby involving MYO18A more directly in this process.

In summary, we postulate that MYO18A acts with Lurap1, p190RhoGEF and Golgin45 in regulating myofiber integrity through at least two related mechanisms. First, this complex regulates the organization of actin skeleton in myofibers. Second, it regulates the normal morphology of the Golgi apparatus, thus facilitating processing, sorting and trafficking of proteins. The link between MYO18A function and myofiber stability should help to elucidate potential human diseases related to unconventional myosins.

## Material and Methods

### Embryos and microinjections

Zebrafish embryos were maintained at 28.5 °C and staged according to standard criteria^52^. Microinjections were performed at one-cell stage using a PLI-100A pico-liter injector (Harvard Apparatus).

The animal experiments were approved by the Experimental Animal Committee of Shandong University, and performed in accordance with the regulations and guidelines.

### Expression constructs

The EGFP-tagged chicken MYO18A PDZ domain and mouse Golgin45 coding sequence were cloned in pEGFP-C2 vector after the GFP coding sequence in *Eco*R I and *Sal* I, and *Hind* III and *Sal* I sites, respectively. The N-terminal myc-tagged full-length mouse MYO18A was cloned in *Not* I and *Hind* III sites of the modified pEGFP-C2 vector with GFP sequence replaced by a myc-coding sequence. The chicken Lurap1 coding region and p190RhoGEF carboxyl-terminal region corresponding to amino acids 951 to 1390 after the pleckstrin homology (PH) domain were also cloned in the modified pEGFP-C2 vector in *Eco*R I and *Sal* I sites. All constructs were sequenced before use.

### Yeast two-hybrid screen

The cDNA encoding the PDZ domain of chicken MYO18A (194–338aa) was amplified from chicken inner ear cDNA and cloned into *pBD-GAL4Cam* (Stratagene) to express the bait protein. Yeast strain *AH109* (Clontech) was transformed with this bait plasmid, followed by transformation with a chicken inner ear cDNA library in *pAD-GAL4*-based phagemid^53^. For screening, *HIS3* and *ADE2* were used as the primary reporter genes in the presence of 2.5 mM 3-amino-1,2,4-triazole. Totally, 5 × 10^7^ transformants were selectively screened and the positive colonies were tested with the third reporter gene *lacZ*. The *pAD-GAL4*-based vectors in triple-positive yeast colonies were recovered and cDNA inserts were sequenced.

### Co-immunoprecipitation

HEK293T cells were transfected with different plasmids using Lipofectamine 2000 (Invitrogen) and cultured for 24 hours. The cells were then washed with phosphate-buffered saline (PBS) at room temperature and lysed in ice-cold lysis buffer composed of 150 mM NaCl, 50 mM Tris-HCl, pH 7.5, 1% (vol/vol) Triton X-100, 1 mM PMSF, and 1 X protease inhibitor cocktail (Sigma-Aldrich). Monoclonal anti-myc antibody agarose beads were used for immunoprecipitation according to the manufacturer’s recommendation (Pierce). After 2 hours of incubation at 4 °C, immunoprecipitated proteins were washed five times with high salt lysis buffer (500 mM NaCl) and separated by polyacrylamide gel electrophoresis, then transferred to nitrocellulose membrane, probed with anti-GFP antibody (Roche) or anti-myc antibody (Santa Cruz Biotechnology) and detected with an Odyssey Infrared Imaging System (LI-COR Biosciences).

### Morpholinos (MOs)

The gene-specific MOs for *myo18aa* and *myo18ab* as well as the *myo18ab* control MO with 5 nucleotide mismatches have been previously described^11^. *p190RhoGEF*MO (5′-GCTGGATGAAATGACTCACTGTGTT-3′) designed over the splicing acceptor site of exon 5, *lurap1*MO (5′-CACAGGTATTATTACTCTCCTCCAT-3′) and *Golgin45*MO (5′-CCTTTTCCACTTCCAGAACACAACA-3′) designed over the translation initiation site were synthesized by Gene Tools. All MOs were diluted in sterile water and stored at −20 °C as small aliquots.

### *In situ* hybridization

Whole-mount *in situ* hybridization was performed according to standard protocol^54^. The cDNAs corresponding to zebrafish *lurap1, p190RhoGEF* and *Golgin45* were amplified by PCR according published sequences and cloned in pGEM-T vector (Promega). Muscle-specific *mhc* and *myo18ab* probes were previously described^11^. Antisense probes were labelled using digoxigenin-11-UTP and appropriate RNA polymerase (Roche). After *in situ* hybridization, some embryos were embedded in Paraplast Plus (Sigma-Aldrich) and sections of 20 μm thickness were cut and examined under a Leica DM2500 microscope.

### Myoblast cell culture

Embryos were injected with CoMO or gene-specific MOs at one-cell stage and were cultured to 24 hpf. The somites were surgically dissected and dissociated through incubation in Ca^2+^/Mg^2+^-free Ringer’s medium followed by treatment with α-chymotrypsin (Sigma-Aldrich). Completely dissociated cells were transferred to a CELLVIEW glass bottom cell culture dish (Grener Bio One) previously coated with laminin (Sigma-Aldrich) at a concentration of 1.5 μg/cm^2^ and cultured at 28.5 °C in 0.66 X Leibovitz L-15 medium (Invitrogen) supplemented with 10% fetal bovine serum and penicillin-streptomycin for 12 to 24 hours.

### Immunofluorescence and confocal microscopy

Whole embryos and cultured cells were fixed overnight in 4% paraformaldehyde in PBS at room temperature. After washing in PBS with 0.1% Tween-20 (PBT) and blocking in 10% sheep serum in PBT, they were incubated for 1 hour in the primary antibody followed by fluorescein-conjugated secondary antibody. After several washes in PBT, the embryos or cells were mounted onto CELLVIEW glass bottom cell culture dishes in Mowiol and analyzed using a Zeiss LSM700 confocal microscope. Antibodies against MHC of slow muscle (F59), MLC of fast muscle (F310), muscle MHC II (MF20), and dystrophin (7A10) were from Developmental Studies Hybridoma Bank (University of Iowa). The antibody against GM130 (a cis-Golgi matrix protein) was from Sigma-Aldrich. Staining with DAPI and TRITC-phalloidin (all from Sigma-Aldrich) were performed after incubation of antibody.

### Histology

Embryos were fixed by 4% paraformaldehyde for 2 hours at room temperature, they were dehydrated in a graded series of ethanol and embedded in polyethylene glycol distearate 400 (Sigma-Aldrich). Sections of 10 μm thickness were cut and stained with hematoxylin and eosin.

### Test of movement ability

Individual embryo at 72 hpf was placed in a culture dish under a Olympus SZX16 stereomicroscope, a fine needle mounted on a micromanipulator was used to gently touch the embryo. The behaviour of the embryo was scored at 10 and 30 ms following the stimulation under a CCD camera (Olympus DP72).

## Additional Information

**How to cite this article**: Cao, J.-M. *et al*. Identification of novel MYO18A interaction partners required for myoblast adhesion and muscle integrity. *Sci. Rep.* **6**, 36768; doi: 10.1038/srep36768 (2016).

**Publisher’s note:** Springer Nature remains neutral with regard to jurisdictional claims in published maps and institutional affiliations.

## Supplementary Material

Supplementary Information
