# Supplementary material for: Identification of novel MYO18A interaction partners required for myoblast adhesion and muscle integrity
Source: Sci Rep. 2016 Nov 8;6:36768. doi: 10.1038/srep36768 (PMC5099880; doi:10.1038/srep36768)
Supplement: Supplementary Information [file srep36768-s1.pdf]

Identification of novel MYO18A interaction partners required for myoblast adhesion and muscle integrity

Jian-Meng Cao[1#], Xiao-Ning Cheng[1#], Shang-Qi Li[1], Stefan Heller[2], Zhi-Gang Xu[1*] and De-Li Shi[1,3*]

[1]School of Life Sciences, Shandong University, Jinan 250100, China

[2]Department of Otolaryngology-Head & Neck Surgery; Institute for Stem Cell Biology and Regenerative Medicine, Stanford University School of Medicine, Stanford, CA 94305, USA

[3]Sorbonne Universités, UPMC Univ Paris 06, CNRS, Biologie du développement, Institut de Biologie Paris Seine (LBD - IBPS), 75005 Paris, France

[*]These authors contributed equally to this work.

Correspondence and requests for materials should be addressed to Z.G.X. (email: xuzg@sdu.edu.cn) or D.L.S. (email: de-li.shi@upmc.fr).

Running title: MYO18A partners in myofiber integrity

Supplementary Figures

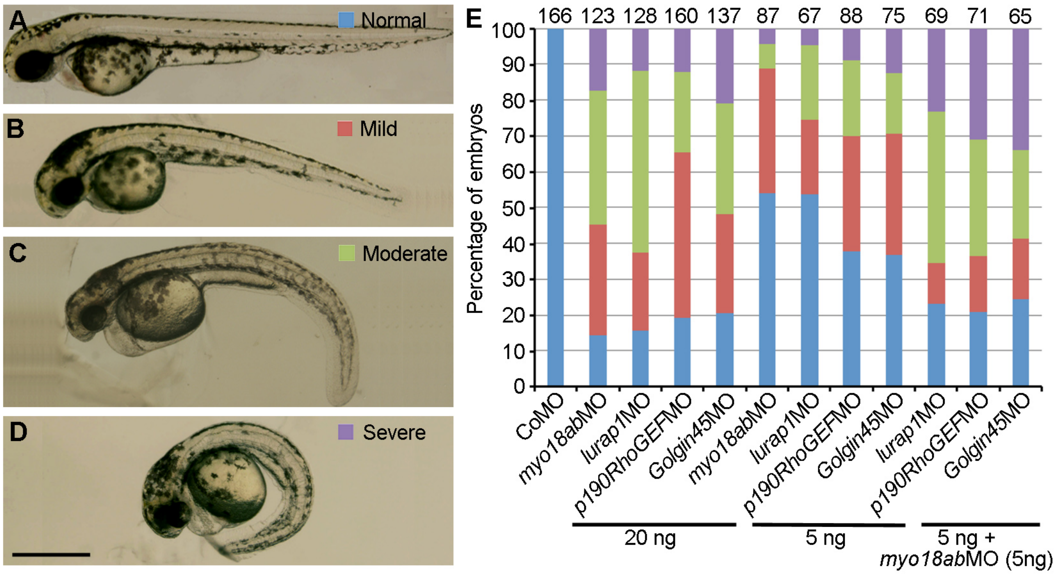

Supplementary Figure 1. Knockdown of *myo18ab*, *lurap1*, *p190RhoGEF* and *Golgin45* affects embryonic development. (A-D) Representative phenotypes from control and indicated morphant embryos at 48 hpf. (E) Statistical analysis of different phenotypes following injection of various MOs, as indicated. Numbers on the top of each column indicate total embryos blindly scored from four independent experiments. Scale bar: (A-D) 250 μm.

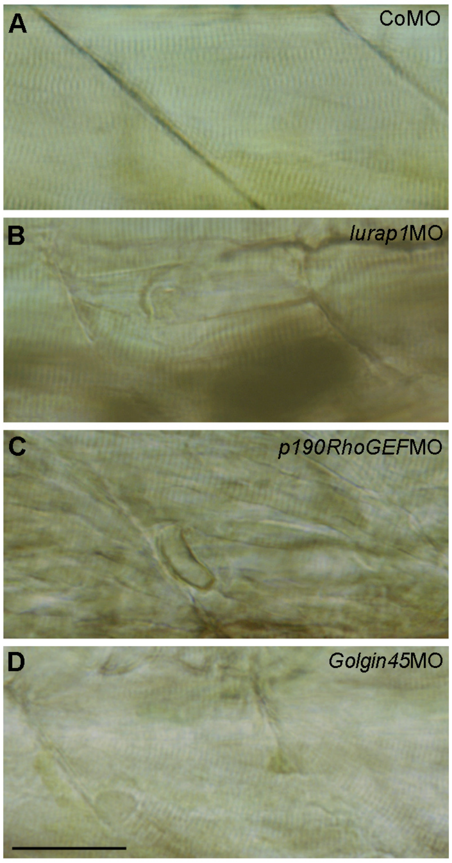

Supplementary Figure 2. Disorganization of myosepta and sarcomere structure in *lurap1*, *p190RhoGEF* and *Golgin45* morphants. (A-D) Differential interference contrast images of myofiber organization in a CoMO-, a *lurap1a*MO-, a *p190RhoGEF*MO- and a *Golgin45*MO-injected embryo at 96 hpf, as indicated. Scale bar: 20 µm.

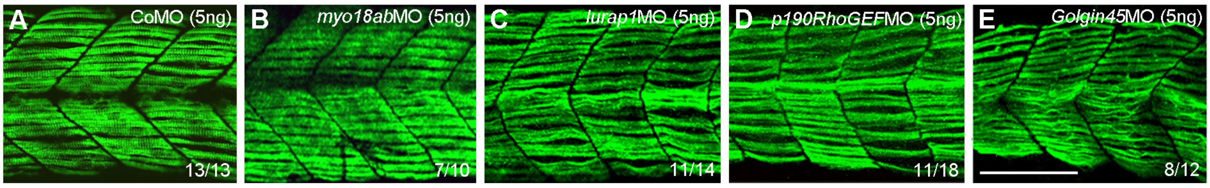

Supplementary Figure 3. Dose-dependent effect of different MOs on myofiber organization. (A-E) Injection of a low amount (5 ng) of each of the indicated MOs mildly affects somite structure and myofiber integrity. Scale bar: 100 µm.

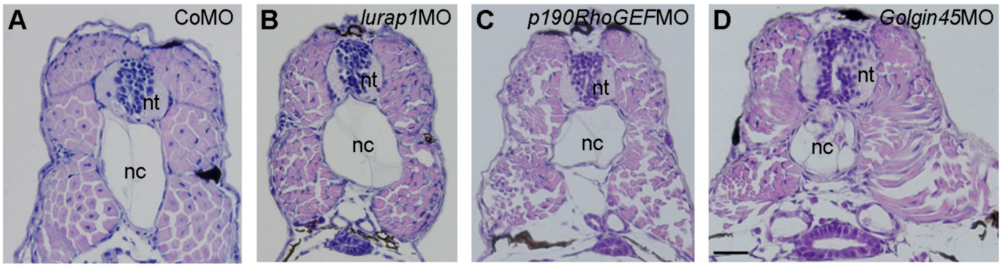

Supplementary Figure 4. Knockdown of *lurap1*, *p190RhoGEF* and *Golgin45* affects the integrity of deep muscle cells. (A-D) Histological sections of indicated embryos at 96 hpf showing deep fast muscle myofiber organization. Scale bar: 50 µm.

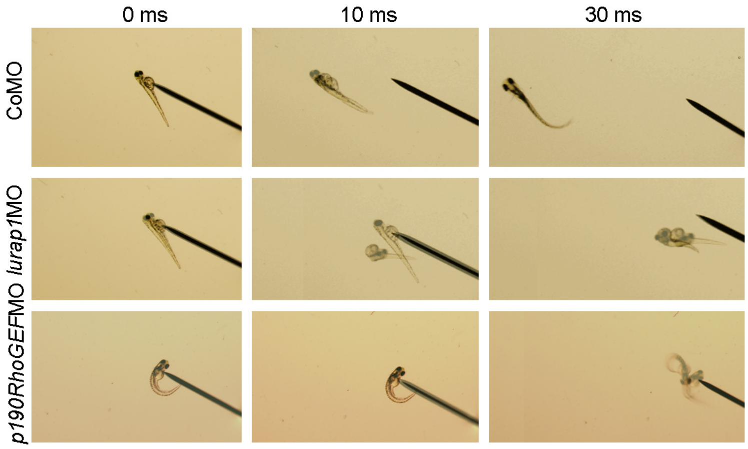

Supplementary Figure 5. Movement defects in *lurap1* and *p190RhoGEF* morphant embryos. Test of "escape response" on indicated embryos at 72 hpf.
